# Supplementary material for: Circadian Variation of Root Water Status in Three Herbaceous Species Assessed by Portable NMR
Source: Plants (Basel). 2021 Apr 16;10(4):782. doi: 10.3390/plants10040782 (PMC8073897; doi:10.3390/plants10040782)
Supplement: Supplementary file 1 [file plants-10-00782-s001.zip › plants-1164804-supplementary.pdf]

# Supporting information for:

# Circadian variation of root water status in three herbaceous species assessed by portable NMR

**Magali Nuixe[1,2,3], Amidou Sissou Traoré[1,2*], Shannan Blystone[1,2,3], Jean-Marie Bonny[1,2], Robert Falcimagne[3], Guilhem Pagés[1,2] and Catherine Picon-Cochard[3,*]**

1 INRAE, UR QuaPA, 63122 Saint-Genès Champanelle France; magali.nuixe@inrae.fr (M.N.) https://orcid.org/0000-0003-0960-8525, amidou.traore@inrae.fr (A.S.T.) ) https://orcid.org/0000-0002-3574-3475, shannan.blystone@inrae.fr (S.B.) https://orcid.org/0000-0002-8494-2411, jean-marie.bonny@inrae.fr (J-M. B.) https://orcid.org/0000-0003-2858-7459, guilhem.pages@inrae.fr (G.P.) https://orcid.org/0000-0001-9368-5237

2 AgroResonance, INRAE, 2018. Nuclear Magnetic Resonance Facility for Agronomy, Food and Health, doi: 10.15454/1.5572398324758228E12

3 Université Clermont Auvergne, INRAE, VetAgro Sup, UREP, 63000 Clermont-Ferrand, France ; robert.falcimagne@inrae.fr (R. F.),catherine.picon-cochard@inrae.fr (C.P-C.) https://orcid.org/0000-0001-7728-8936

* Correspondence: amidou.traore@inrae.fr +33(0) 4 73 62 46 29 (A.S.T.); catherine.picon-cochard@inrae.fr +33(0) 4 43 76 16 15 (C.P-C.)

## Figure S1: CPMG decay curves of each species

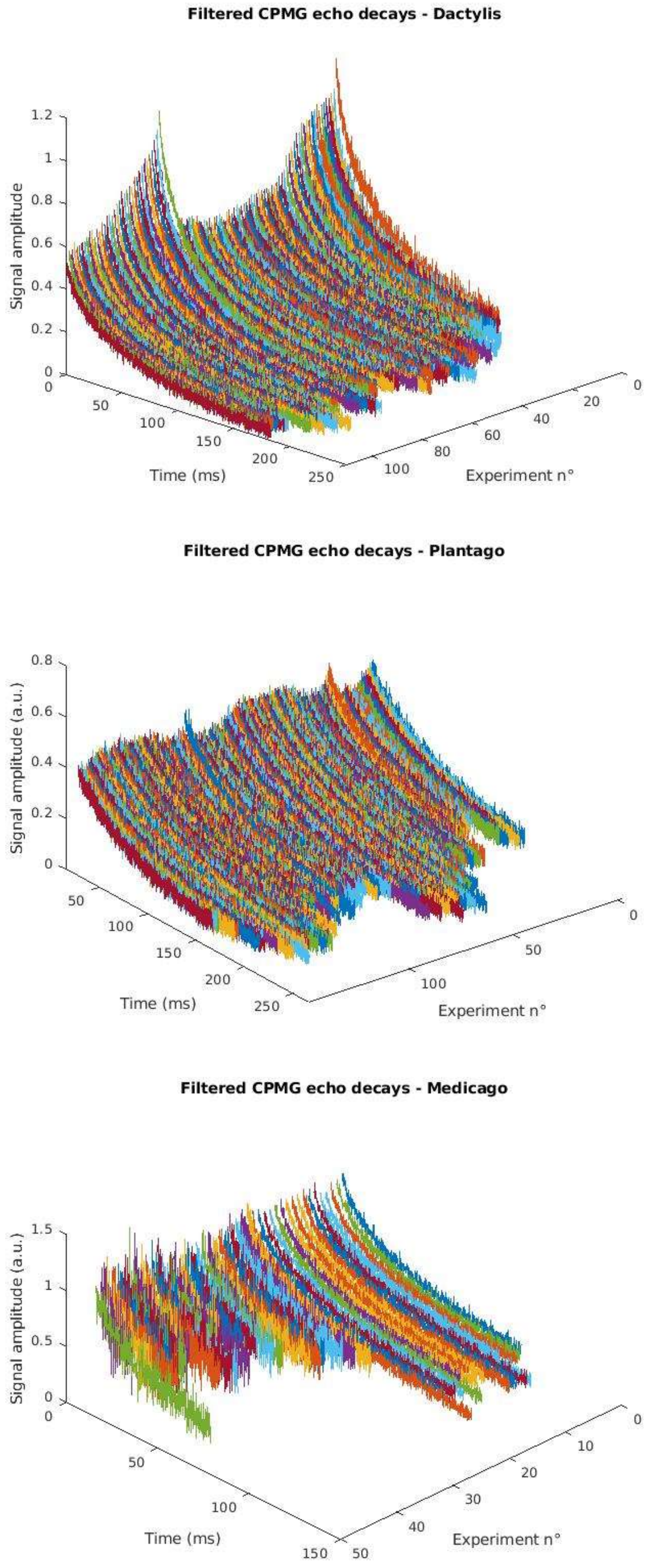


**Figure S1.** Filtered CPMG decay curves of *D. glomerata* (top), *P. lanceolata* (middle), and *M. sativa* (bottom)

**Methods S1. Processing of the transverse relaxation decay curves**

A representation of the transversal decay data obtained with the CPMG pulse sequence is presented in Fig S2A. Before any ILT analysis, data were first preprocessed by a) removing the first 10 points which were highly affected by both $B_0$ and $B_1$ inhomogeneities, i.e., high oscillations, and b) removing the last noisy points, i.e. all points after the first echo with an amplitude less than or equal to zero.

Assuming that the measured echo decays stands as a superposition of exponential decays, the signal can be expressed as:

$$S(t_i) = \sum_{j=1}^{M} A_j \mathrm{e}^{-t_i/_{T2j}}, \qquad i = 1,2,\dots,N \qquad [1]$$

where $t_i$ is the measurement time, M is the number of exponential components, N is the total number of data points, and $A_j$ is the relative amplitude for each partitioned $T_2$ time, T2j.

In the present study, the multiexponential analysis was performed using an in-house Matlab® implementation of the non-negative least squares (NNLS) algorithm [42]. To recover the M number of exponential components, their amplitudes $A_j$ and their relaxation times $T2_j$, the NNLS algorithm was fed with a large M (i.e., 100) number of $T2_j$ values logarithmically spaced from 1 ms to 1000 ms. To better reflect the continuous distribution of water commonly found in biological systems, an extra regularization constraint was added to smooth the estimated discrete distribution of $A_j$ provided by NNLS. The regularized NNLS solution was then a set of amplitudes $A_j$ that minimize the misfit:

$$\sum_{i=1}^{N} \left| \sum_{j=1}^{M} A_{ij} S_j - y_i \right|^2 + \mu \sum_{j=1}^{M} \left| S(T_{2j}) \right|^2, \mu \geq 0 \qquad [2]$$

where the Lagrangian term μ is automatically calculated using the cross-validation approach [43].

**Figure S2. Flow of NNLS inversion of the transversal relaxation decay curves.**

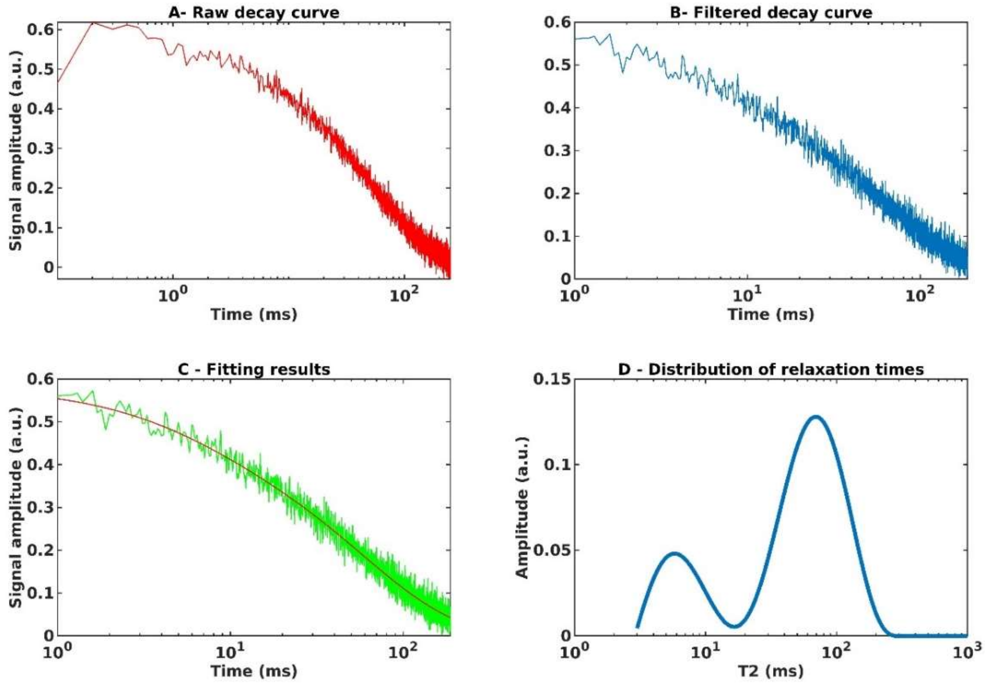


**Figure S2.** Flow of NNLS inversion of the transversal relaxation decay curves. A : Full transversal relaxation decay curve. B : Filtered decay after removal of the first 10 echo amplitude signals and the last and only noisy data points. C : Filtered decay (green) along with the fitted model (red) after NNLS analysis. D : The resulting distribution of $T_2$ relaxation provided by NNLS.

**References**


42. Lawson, C.L.; Hanson, R.J. *Solving Least Squares Problems*; Classics in applied mathematics, 1995; ISBN 0-89871-356-0.

43. Bjarnason, T.A.; McCreary, C.R.; Dunn, J.F.; Mitchell, J.R. *Quantitative $T_2$ Analysis: The Effects of Noise, Regularization, and Multivoxel Approaches.* Magn Reson Med 2010, 63, 212–217, doi:10.1002/mrm.22173.
